# Prevalence, Appropriateness, and Outcomes of Colistin Use in Multidrug-Resistant *Pseudomonas aeruginosa* Infections: Insights from Hospital Data

**DOI:** 10.3390/medicina61071275

**Published:** 2025-07-15

**Authors:** Rana K. Abu-Farha, Savana Sobh, Khawla Abu Hammour, Feras Darwish El-Hajji, Sireen A. Shilbayeh, Rania Itani

**Affiliations:** 1 Clinical Pharmacy and Therapeutics Department, Faculty of Pharmacy, Applied Science Private University, Amman P.O. Box 11937, Jordan; savanasobh@gmail.com (S.S.); f_elhajji@asu.edu.jo (F.D.E.-H.); 2 Department Biopharmaceutics and Clinical Pharmacy, Faculty of Pharmacy, The University of Jordan, Amman 11942, Jordan; k.hammour@ju.edu.jo; 3 Department of Pharmacy Practice, College of Pharmacy, Princess Nourah bint Abdulrahman University, P.O. Box 84428, Riyadh 11671, Saudi Arabia; ssabdulrahim@pnu.edu.sa; 4 Pharmacy Practice Department, Faculty of Pharmacy, Beirut Arab University, Beirut P.O. Box 11-5020, Lebanon; r.itani@bau.edu.lb

**Keywords:** *Pseudomonas aeruginosa*, colistin, antimicrobial resistance, multidrug-resistance, Jordan

## Abstract

*Background and Objectives*: This study aimed to assess the prevalence of colistin prescriptions among patients with multidrug-resistant (MDR) *Pseudomonas aeruginosa* (*P. aeruginosa)* infections admitted to a tertiary teaching hospital in Jordan. Additionally, the study evaluated the appropriateness of colistin prescriptions and assessed resistance levels of this strain. *Materials and Methods*: In this retrospective study, adult patients who were infected with *MDR P. aeruginosa* and were admitted to Jordan University Hospital between January 2018 and March 2024 were included. Data on demographics, clinical characteristics, sources of infection, antibiotic therapy, and clinical outcomes were collected. *Results*: Out of the 85 patients who met the inclusion criteria for having MDR *P. aeruginosa*, colistin was administered to 16 patients (18.8%). Notably, approximately two-thirds (68.7%) of the isolates from patients who received colistin were classified as extensively drug-resistant (XDR). Among the isolates, 15 out of 16 (93.8%) were resistant to both ciprofloxacin and imipenem. Among the patients requiring colistin, five (31.3%) discontinued therapy, while two (12.5%) remained on colistin despite the availability of safer alternatives. No significant difference was observed in 30-day all-cause mortality between patients treated with colistin (0%) and those who were not (4.3%, *p* = 1.00). Similarly, the incidence of acute kidney injury did not differ significantly between the colistin group (0%) and the non-colistin group (*p* = 1.00). No significant difference was found in the hospital stay between colistin-treated patients (median 10.5 days, IQR [5.0–14.0]) and those not treated with colistin (median 13.0 days, IQR [7.0–21.0]), (*p* = 0.22). *Conclusions*: This study demonstrated that colistin was selectively initiated in high-risk patients, particularly those with XDR *P. aeruginosa*. However, its inappropriate continuation despite safer alternatives, as well as its discontinuation when no other options existed, raise concerns about antibiotic de-escalation practices. Interestingly, no significant differences in mortality or acute kidney injury were observed between patients who were treated with colistin and those who were not. These findings emphasize the need for antimicrobial stewardship programs and highlight the importance of large-scale trials to evaluate colistin’s efficacy and safety in MDR infections.

## 1. Introduction

Antimicrobial resistance has become a global serious problem due to the increasing number of resistant strains of pathogens—which can be called ‘superbugs’—at a faster rate than the development of new antimicrobials [1,2]. MDR bacteria is defined as “non-susceptibility to at least one agent in three or more antimicrobial categories” [2]. The burden of antimicrobial resistance is becoming inevitable, which includes loss of effective antibiotics, reduced therapeutic choices, increased rate of hospitalization with all related costs and loss of productivity, morbidities, public health crises and healthcare system collapse, and high risk of mortality [1,2].

*Pseudomonas aeruginosa* (*P. aeruginosa*) is an opportunistic highly mutated bacterium that demands special attention upon treatment due to the great therapeutic challenge it exerts. While MDR infections by *P. aeruginosa* are dramatically increasing in hospital inpatients, the choices of effective antibiotics are becoming limited with time. MDR *P. aeruginosa* are typically found in isolates taken from intensive-care unit (ICU) patients and patients on mechanical ventilation [3,4].

*P. aeruginosa* is characterized by developing complex resistance mechanisms towards a wide range of antibiotics. Such mechanisms can be imported, and they are chromosomally regulated and co-regulated. MDR *P. aeruginosa* isolates can be resistant to a wide range of antibiotics, including the broad pharmacological families of β-lactams, fluoroquinolones, and aminoglycosides [3,4]. The mutations of *P. aeruginosa* are believed to develop resistance against even highly effective antibiotics such as colistin [4,5].

Colistin belongs to polymyxins antibiotics, a group of basic peptides active against Gram-negative bacteria. Its mechanism of action includes disrupting the bacterial cell membrane and also inactivating endotoxins [6]. It is classified as a ‘reserve’ antibiotic according to the World Health Organization AWaRe classification of antibiotics in 2023 [6]. In Jordan, colistin is listed on the Essential Medicines List; however, the antibiotic prescribing and dispensing regulations with its high cost keep the drug unaffordable to Jordanian population [7].

Colistin antibiotic is considered a relatively narrow-spectrum antibiotic with clinically proved efficacy in eradication of *P. aeruginosa* infections [7,8]. Hitherto, sensitivity of *P. aeruginosa* blood and respiratory isolates is considered among the highest [4]. Yet, this antibiotic is recommended to be reserved for life-threatening infections, usually by MDR bacteria due to its nephrotoxicity [9,10].

In Jordan, nearly half of the *P. aeruginosa* isolates (47.1%) have been reported as MDR, further underscoring the importance of evaluating how last-line therapies such as colistin are being utilized [11]. Recently, resistance to colistin has been increasing due to inappropriate prescription patterns, threatening its use as a reserve antibiotic. This study aimed to evaluate the prevalence and appropriateness of colistin prescriptions among patients with multidrug-resistant *Pseudomonas aeruginosa* infections admitted to a tertiary teaching hospital in Jordan. The outcomes of this research are expected to contribute to a better understanding of current prescribing patterns and inform future efforts related to antimicrobial stewardship and the rational use of reserve antibiotics in clinical practice.

## 2. Materials and Methods

### 2.1. Study Design and Patient Selection

This retrospective study was carried out at Jordan University Hospital (JUH), which is the first teaching tertiary health institution in Jordan. This study comprised adult patients, aged 18 and up with MDR P. aeruginosa who were admitted to the hospital between January 2018 and March 2024 [12]. Exclusion criteria included incomplete medical records and patients with polymicrobial infections.

### 2.2. Data Collection

The study included a comprehensive review of electronic medical records for all eligible patients. Relevant data were extracted using a pre-prepared data collection form, covering a range of clinical and demographic information. This included patient age, gender, insurance coverage, admission date, total duration of hospitalization, and department of admission. Data on the site and source of infection acquisition (community-acquired or hospital-acquired), comorbid medical conditions, and any ICU admissions were also collected. The records provided details on specimen types and retrieval dates, as well as information about the empiric antibiotics administered, including initiation date, type, route, dosage, and duration. Additionally, culture and susceptibility test results for various antimicrobial agents were gathered. The definitive antibiotics used were also identified. The study involved documenting any cases of nephrotoxicity or neurotoxicity, as well as recording the 30-day all-cause hospital mortality.

### 2.3. Antimicrobial Susceptibility Testing and Resistance Classification

The identification of *P. aeruginosa* isolates was conducted using a combination of phenotypic and biochemical tests. Initial identification was based on Gram staining, oxidase, and catalase tests, followed by confirmation using the VITEK 2 system (bioMérieux, Marcy-l’Étoile, France). Clinical specimens were inoculated onto blood agar and MacConkey agar and incubated at 37 °C for 48 h to facilitate bacterial growth and isolation.

Antimicrobial susceptibility testing (AST) was conducted using the disk diffusion method in accordance with the Clinical and Laboratory Standards Institute (CLSI) recommendations. The susceptibility profiles of *P. aeruginosa* isolates were evaluated against a panel of antibiotics including amikacin, gentamicin, aztreonam, cefepime, ceftazidime, ceftazidime–avibactam, ceftolozane–tazobactam, piperacillin–tazobactam, ciprofloxacin, colistin, imipenem, and meropenem. Susceptibility results were interpreted based on CLSI guidelines [9,13]. To ensure accurate and standardized susceptibility assessment, the minimum inhibitory concentration (MIC) for colistin was determined using the broth dilution method, as recommended by CLSI and the European Committee on Antimicrobial Susceptibility Testing (EUCAST) [9,13].

Based on the observed resistance patterns, the isolates were classified into three categories: MDR isolates, which are resistant to at least one agent in three or more antibiotic classes; extensively drug-resistant (XDR) isolates, which are resistant to at least one agent in all but two or fewer antibiotic classes; and pan-drug-resistant (PDR) isolates, which are resistant to all antibiotics typically used for treatment [2].

Finally, for patients who received colistin, all potential alternatives were identified based on the antibiogram profiles. This evaluation focused on antibiotics that could serve as effective treatment options according to the susceptibility data.

### 2.4. Statistical Analysis

Data were analyzed using IBM SPSS Statistics for Windows, Version 26.0 (IBM Corp., Armonk, NY, USA). Continuous variables were presented as median and interquartile range (IQR), while categorical variables were presented as frequencies and percentages. Normality checks for continuous variables were conducted using the Shapiro–Wilk test. Fisher’s exact test was used to compare categorical outcomes between patients who received colistin and those who did not, as the percentage of cells with expected cell counts exceeded 20%, making it more appropriate than the chi-square test. On the other hand, the Mann–Whitney U test was used to compare the difference in continuous outcomes (length of stay) between patients who received colistin and those who did not, since this outcome was found to be not normally distributed. Results with a *p* ≤ 0.05 were regarded as significant in statistical terms.

## 3. Results

In this study, 85 patients who met the inclusion criteria were included, as shown in Table 1. The patients were almost equally distributed by gender, with 44 females (51.8%) and 41 males (48.2%). Patients had a median age of 53 years (IQR [38.0–66.0]). All patients (n = 85, 100%) were insured, and 68 patients (80.0%) had chronic medical conditions. Regarding the duration of hospitalizations, the results indicated that patients had a median hospital stay of 12 days, with an IQR of [6 to 20] days.

Regarding the original specimen sources, the most common were urine (n = 32, 37.6%), followed by skin/soft tissue (n = 19, 22.4%), and sputum (n = 17, 20.0%). All specimens were retrieved from patients before the initiation of empiric antibiotics (n = 85, 100%). Most infections were community-acquired (n = 71, 83.5%) rather than hospital-acquired (n = 14, 16.5%). The majority of infections were localized (n = 77, 90.6%), with urinary tract infections being the most common (n = 32, 37.6%), followed by respiratory tract infections (n = 27, 31.8%). A smaller proportion of patients required ICU admission (n = 16, 18.8%). In terms of 30-day all-cause mortality, 82 patients (96.5%) survived, while 3 patients (3.5%) died.

Among the study cohort, all patients (n = 85, 100%) received empiric antibiotics. According to Table 2, 80.0% (n = 68) received combination empiric therapy, while 20.0% (n = 17) were treated with monotherapy. Colistin was prescribed in 18.8% of cases (n = 16), and it was exclusively administered intravenously (n = 16, 100%), with no instances of nebulized colistin. Among the patients receiving colistin, 14 infections (87.5%) were community-acquired, while 2 (12.5%) were hospital-acquired. The sources of infections for patients receiving colistin were as follows: eleven (68.8%) had urinary tract infections, four (25.0%) had respiratory tract infections, and one (6.3%) had a surgical site infection. All of the patients with these infection sources received combination empiric therapy, except for one urinary tract infection patient, who was treated with monotherapy. The most commonly used concomitant antibiotics were imipenem–cilastatin (n = 11, 68.8%), piperacillin–tazobactam (n = 5, 31.3%), and vancomycin (n = 4, 25.0%). This reflects clinical practice to broaden antimicrobial coverage in severe or resistant infections while awaiting culture results.

Based on Table 3, all *P. aeruginosa* isolates were classified as MDR, with 11 out of the 16 (68.7%) being XDR. These isolates exhibited high resistance rates to several critical antibiotics. Specifically, 15 out of 16 isolates (93.75%) were resistant to ciprofloxacin, 14 out of 16 (87.5%) were resistant to both gentamicin and ceftazidime, and 14 out of 16 (87.5%) were resistant to meropenem, while 15 out of 16 (93.75%) were resistant to imipenem.

It is important to note that resistance testing for colistin was conducted for only 8 out of the 16 patients with *P. aeruginosa* infections. None of the tested isolates were resistant to colistin.

Table 4 provides an overview of potential alternative antibiotics to colistin for treating *P. aeruginosa* infections, based on the antibiogram profiles of the 16 patients. Evaluating these alternatives is important to ensure colistin is used only when necessary and to guide antibiotic stewardship. The data indicate that several patients had possible safer alternatives to colistin, such as aztreonam, piperacillin–tazobactam, cefepime, ceftazidime, and ceftazidime–avibactam. Specifically, patients 1, 2, 4, 14, and 15 had multiple effective and safer options based on their susceptibility profiles.

Following the culture and susceptibility data, colistin was continued as definitive therapy for only five patients (31.3%) (Table 4). Among these, three patients (3, 8, and 16) were correctly maintained on colistin due to the absence of viable alternative antibiotics based on their antibiogram. However, the other two patients continued on colistin despite the availability of safer alternatives. In contrast, colistin therapy was discontinued for five patients (6, 7, 9, 12, and 13), despite their antibiogram showing resistance to all other tested agents, leaving them on antibiotics to which they were resistant.

Table 5 shows that the use of colistin as empiric treatment resulted in no significant difference in 30-day all-cause mortality compared to patients who did not receive colistin empirically (0% vs. 4.3%, *p* = 1.00). Additionally, there was no significant difference in hospital stay duration between patients treated with colistin (median 10.5 days, IQR [5.0–14.0]) and those not treated with colistin (median 13.0 days, IQR [7.0–21.0]), (*p* = 0.22). Furthermore, there was no significant difference in the incidence of acute kidney injury between the two groups; none of the patients receiving colistin experienced this complication, while 4.3% of those not receiving colistin did (*p* = 1.00). This suggests that, despite its known nephrotoxic potential, colistin did not lead to a higher rate of acute kidney injury in this cohort. No cases of neurotoxicity were observed in either group. This finding indicates that colistin use, in this cohort, did not significantly alter clinical outcomes or increase adverse events.

## 4. Discussion

The primary aim of this study was to evaluate the prevalence, appropriateness, and treatment outcomes of colistin therapy in patients with MDR *P. aeruginosa* infections. Colistin is classified as a reserve antibiotic and is typically prescribed when there is strong suspicion or confirmation of MDR *P. aeruginosa* infections [6]. The judicious use of colistin is crucial for minimizing the unnecessary administration of last-line antibiotics and preventing further resistance development [14].

Due to its toxicity and limited efficacy compared to other antimicrobial agents, colistin is primarily reserved as a salvage therapy. Its routine use is generally discouraged, as it is considered a last resort. According to the CLSI, colistin should be restricted to salvage therapy for infections caused by MDR *P. aeruginosa*, *Acinetobacter baumannii*, or carbapenem-resistant Enterobacterales (CRE), particularly when no other active antimicrobials are available. However, in certain high-risk scenarios, its use may be warranted. Critically ill patients with invasive infections and a high likelihood of infection with XDR pathogens may require colistin therapy. Furthermore, its administration may be justified in cases of ventilator-associated pneumonia (VAP) in healthcare settings with a high prevalence of MDR *P. aeruginosa*, *Acinetobacter baumannii*, or CRE, as well as in severe nosocomial infections where carbapenem resistance is widespread, and no safer alternatives exist. Given colistin’s significant nephrotoxicity, its use should be strictly confined to situations where no other effective treatment options are available [9,10,15,16,17].

In this study, colistin was utilized in a select group of patients as part of their early empirical treatment regimen. Of the total cohort, 16 patients (18.8%) received colistin empirically. Notably, 93.8% of patients who received colistin were also administered additional antibiotics, particularly imipenem–cilastatin and piperacillin–tazobactam. This combination raises concerns, as previous studies have shown that colistin combined with other antibiotics was not superior to colistin monotherapy in terms of efficacy [18,19,20]. Furthermore, the use of colistin alongside other broad-spectrum antibiotics may lead to unnecessary resource utilization, increased risk of antibiotic resistance, and contribute to the development of adverse side effects [20]. Our results align with those of Giacobbe et al., who found that in 80% of instances, intravenous colistin was used alongside another anti-MDR Gram-negative bacterial drug. The agents included in these combinations were carbapenems, aminoglycosides, tigecycline, rifampicin, ceftazidime–avibactam, and ceftolozane–tazobactam [21]. This combination therapy was mainly used for treating severe lower respiratory tract infections and bloodstream infections caused by carbapenem-resistant organisms. The use of the combination may be driven by concerns about the suboptimal pharmacokinetics of colistin or the desire to achieve synergy or additive effects with other agents, despite limited evidence supporting its efficacy in reducing clinical failure in MDR infections [21].

The data shows that the majority of isolates exhibit high resistance rates to several critical antibiotics, with 93.8% of isolates resistant to ciprofloxacin and imipenem, and 87.5% resistant to gentamicin, ceftazidime, and meropenem. This indicates that colistin is primarily used as a last-resort treatment for often life-threatening infections caused by MDR *P. aeruginosa* [22], a practice recommended by the WHO [6].

Colistin susceptibility testing was performed on only 8 out of the 16 patients (50%) in our study, with none of the tested isolates showing resistance. In comparison, Giacobbe et al. found that colistin susceptibility testing had been conducted on 183 out of 198 (92%) causative isolates in their cross-sectional study from Italy, highlighting a much higher testing rate compared to our findings [21]. Appropriate AST is essential for confirming the susceptibility of bacterial isolates to colistin and identifying any resistance present [23]. This may help in avoiding the use of ineffective therapies and in guiding the rational use of antibiotics, ensuring that patients receive the most appropriate treatment.

In this study, there are cases where patients continued on colistin as definitive therapy despite the availability of alternative antibiotics. Specifically, two patients were identified as having viable treatment options but remained on colistin. This decision often stems from the severity and urgency of the infection [24]. Additionally, patient-specific factors such as comorbidities, prior treatment responses, and the inability to tolerate alternatives may have a crucial role in continuing colistin despite the availability of alternative antibiotics [25]. However, this raises a concern: while there may be instances where continuing colistin is warranted, no justifying documentation was noted in the patients’ medical files. Also, the retrospective nature of the study makes it difficult to assess the rationale behind these decisions.

Moreover, the study showed that five patients were identified as having no viable alternatives to colistin and yet did not continue colistin therapy. Without effective treatment, these patients may experience longer hospital stays or require additional interventions due to complications from untreated infections [26]. Furthermore, inadequate treatment can contribute to the development of antibiotic resistance, complicating future treatment options [26].

When evaluating 30-day all-cause mortality, there was no significant difference among patients who received colistin empirically versus those who did not (0% vs. 4.3%, *p* = 1.00). This lack of statistical difference suggests that the use of colistin as empiric therapy did not confer a survival benefit, aligning with other studies that indicate colistin’s efficacy is comparable to other treatment regimens in similar settings [27]. However, the small sample size may have hindered the ability to detect substantial mortality differences.

Furthermore, the study revealed no significant difference in the hospital stay between colistin-treated patients and those not treated with colistin, (*p* = 0.22). Notably, none of the patients who received colistin empirically experienced acute kidney injury or neurotoxicity, which counters concerns about its nephrotoxicity and aligns with findings from other studies [25]. Overall, these findings indicate that colistin, when used empirically, did not result in worse outcomes regarding mortality, hospital stay, or adverse events such as acute kidney injury or neurotoxicity.

The study has a few notable limitations. First of all, the small sample size, particularly the limited number of patients receiving colistin restricts the statistical power to detect differences or assess the impact of underlying patient conditions on treatment outcomes. Larger studies are needed to better evaluate these relationships. Second, the retrospective data collection hinders the ability to accurately report adverse effects, particularly nephrotoxicity and neurotoxicity. Additionally, due to the retrospective nature of the study, it was difficult to understand the rationale for physician decisions in prescribing or discontinuing colistin therapy, as not all data were reported within the medical records. Furthermore, since the study was conducted in one tertiary center, this may limit the generalizability of the data to other health institutions. Moreover, one of the main limitations of this study is the use of antibiotics in susceptibility testing of *Pseudomonas* isolates that are not included in the CLSI guidelines (e.g., gentamicin, ceftolozane–tazobactam, and tigecycline). However, these antibiotics were selected for testing based on the treatment protocol at the studied hospital. The lack of adherence to the CLSI guidelines may limit the generalizability of the results. Also, the absence of susceptibility testing for various antibiotics restricts the identification of all viable treatment options available to patients, potentially impacting the selection of appropriate therapies. In addition, although the VITEK 2 system is a widely used and reliable tool for AST, it may have limitations in detecting extremely low bacterial loads, especially in cases of early-stage infections or infections involving biofilms. This limitation could potentially impact the detection of resistance patterns in certain cases and should be considered when interpreting susceptibility results.

Based on the above limitations, it is important to conduct prospective, long-term, multicenter research with larger sample sizes to better understand the appropriateness and safety of prescribing colistin therapy for MDR *P. aeruginosa* patients. This approach will help achieve better control of the different confounders and more accurately evaluate the outcomes of using colistin in this cohort of patients.

## 5. Conclusions

As a last-line salvage therapy, colistin must be used judiciously to minimize unnecessary administration and prevent further resistance development. This study demonstrated that colistin was selectively initiated in high-risk patients, particularly those infected with XDR *P. aeruginosa*. However, its inappropriate continuation despite the availability of safer alternatives, as well as its discontinuation in cases where no other options existed, raise concerns regarding antibiotic de-escalation practices. These findings emphasize the need for robust antimicrobial stewardship programs to ensure optimal therapy selection and prompt tailoring of treatment based on susceptibility data.

## Figures and Tables

**Table 1:** Patient demographics and medical characteristics (n = 85).

Parameter	Median (IQR)	Frequency (%)
Patient gender		
○ Male		41 (48.2)
○ Female		44 (51.8)
Patient age (Years)	53.0 (38.0–66.0)	
Insurance status		
○ Not insured		0 (0)
○ Insured		85 (100)
Having chronic medical conditions		
○ No		17 (20.0)
○ Yes		68 (80.0)
Length of hospital stay	12.0 (6.0–20.0)	
Source of infection acquisition ^		
○ Community acquired		71 (83.5)
○ Hospital acquired		14 (16.5)
ICU admission		
○ No		69 (81.2)
○ Yes		16 (18.8)
Infection spread		
○ Localized		77 (90.6)
○ Non-localized		8 (9.4)
Primary site of infection		
○ Urinary tract infection		32 (37.6)
○ Respiratory tract infection		27 (31.8)
○ Skin/soft tissue		16 (18.8)
○ Surgical site infection		6 (7.1)
○ Ocular infection		2 (2.3)
○ Bloodstream infection		1 (1.2)
○ Osteomyelitis		1 (1.2)
Original specimen		
○ Urine		32 (37.6)
○ Soft tissue/skin		19 (22.4)
○ Sputum		17 (20.0)
○ Others		17 (20.0)
Date of specimen retrieval		
○ Before empiric antibiotic initiation		85 (100)
○ After empiric antibiotic initiation		0 (0)
30-day all-cause mortality		
○ No		82 (96.5)
○ Yes		3 (3.5)

IQR: Interquartile range. ^ Community-acquired infections are those acquired within 48 h of hospital admission, while hospital-acquired infections are those that become evident after 48 h of hospitalization.

**Table 2:** Characteristics of antibiotic therapy and colistin utilization in MDR *Pseudomonas aeruginosa* infections (n = 85).

Parameter	Frequency (%)
Empiric antibiotics received	
○ No	0 (0)
○ Yes	85 (100)
Empiric antibiotic therapy type	
○ Monotherapy	17 (20.0)
○ Combination therapy	68 (80.0)
Colistin received as empiric	
○ No	69 (81.2)
○ Yes	16 (18.8)
Route of colistin administration #	
○ Intravenous	16 (100)
○ Nebulized	0 (0)
Received combination therapy with colistin #	
○ No	1 (6.3)
○ Yes	15 (93.8)
Source of infections in those who received colistin #	
○ Urinary tract infection	11 (68.8)
○ Respiratory tract infection	4 (25.0)
○ Surgical site infection	1 (6.3)
Concomitant antibiotic use alongside colistin ^	
○ Imipenem–cilastatin	11 (68.8)
○ Piperacillin–tazobactam	5 (31.3)
○ Vancomycin	4 (25.0)
○ Levofloxacin	3 (18.8)
○ Meropenem	2 (12.5)
○ Amikacin	2 (12.5)
○ Gentamicin	1 (6.3)
○ Tigecycline	1 (6.3)
○ Ceftazidime	1 (6.3)

# The percentage is calculated among those who received colistin (n = 16). ^ The percentages represent the proportion of patients who received each antibiotic alongside colistin and do not necessarily sum to 100% due to the possibility of multiple antibiotics being used concurrently.

**Table 3:** Antimicrobial susceptibility profile of *Pseudomonas aeruginosa* isolates for patients using colistin (n = 16).

Patient No.	AMK	GEN	TOB	ATM	CFP	CAZ	CAZ-AVI	CEF-TAZ	PIP-TAZ	TIC-CLA	CIP	COL	IMI	MER	TIG	Resistance Pattern
1	S	S	NP	R	R	S	NP	NP	S	NP	S	NP	S	R	NP	MDR
2	S	NP	NP	S	R	S	NP	NP	S	NP	R	NP	R	R	NP	MDR
3	R	R	NP	NP	R	R	NP	NP	NP	NP	R	NP	R	NP	NP	XDR
4	S	R	NP	S	R	R	NP	NP	S	NP	R	NP	R	R	NP	MDR
5	S	S	NP	NP	R	R	NP	NP	NP	NP	R	NP	R	R	NP	XDR
6	R	R	NP	R	R	R	NP	NP	R	NP	R	NP	R	R	NP	XDR
7	R	R	NP	R	R	R	NP	NP	R	NP	R	NP	R	R	NP	XDR
8	R	R	NP	R	R	R	NP	NP	R	NP	R	NP	R	R	NP	XDR
9	R	R	NP	R	R	R	NP	NP	R	NP	R	S	R	R	NP	XDR
10	S	R	NP	NP	R	R	R	R	R	NP	R	S	R	R	NP	XDR
11	S	R	NP	NP	R	R	R	R	R	NP	R	S	R	R	NP	XDR
12	R	R	NP	NP	R	R	NP	NP	R	NP	R	S	R	R	NP	XDR
13	R	R	NP	NP	R	R	R	R	NP	NP	R	S	R	R	NP	XDR
14	S	S	S	NP	S	R	NP	NP	S	R	R	S	R	S	NP	MDR
15	R	S	NP	NP	S	S	S	NP	R	NP	R	S	R	R	NP	MDR
16	R	R	R	NP	R	R	NP	NP	R	R	R	S	R	R	NP	XDR

AMK—Amikacin; GEN—Gentamicin; TOB—Tobramycin; ATM—Aztreonam; CFP—Cefepime; CAZ—Ceftazidime; CAZ-AVI—Ceftazidime–Avibactam; CEF-TAZ—Ceftolozane–tazobactam; PIP-TAZ—Piperacilli–-tazobactam; TIC-CLA—Ticarcillin–clavulanate; CIP—Ciprofloxacin; COL—Colistin; IMI—Imipenem; MER—Meropenem; TIG—Tigecycline. S = Sensitive, R = Resistant, NP = Not Performed, MDR: Multidrug Resistant, XDR: Extensively Drug-resistant.

**Table 4:** Potential alternatives to colistin and ongoing colistin therapy for *Pseudomonas aeruginosa* patients.

Patient No.	Possible Alternatives to Colistin	Continued on Colistin as Definitive Therapy
1	AMK, GEN, CAZ, PIP-TAZ, CIP, IMI	No
2	AMK, ATM, CAZ, PIP-TAZ	Yes
3	None	Yes
4	AMK, ATM, PIP-TAZ	No
5	AMK, GEN	No
6	None	No
7	None	No
8	None	Yes
9	None	No
10	AMK	No
11	AMK	No
12	None	No
13	None	No
14	AMK, GEN, TOB, CEP, PIP-TAZ, MER	No
15	GEN, CEP, CAZ, CAZ-AVI	Yes
16	None	Yes

AMK—Amikacin; GEN—Gentamicin; TOB—Tobramycin; ATM—Aztreonam; CEP—Cefepime; CAZ—Ceftazidime; CAZ-AVI—Ceftazidime–Avibactam; PIP-TAZ—Piperacillin–tazobactam; CIP—Ciprofloxacin; IMI—Imipenem; MER—Meropenem.

**Table 5:** Treatment outcomes for patients treated with colistin as empiric therapy versus those not treated (n = 85).

Outcomes	Use of Colistin (n = 16)	No Use of Colistin (n = 69)	*p*-Value
30-day all-cause mortality, n (%)	0 (0)	3 (4.3)	1.00 #
Acute kidney injury, n (%)	0 (0)	3 (4.3)	1.00 #
Neurotoxicity	0 (0)	0 (0)	NA
Length of hospital stay, median (IQR)	10.5 (5.0–14.0)	13.0 (7.0–21.0)	0.22 ^

# Using Fisher exact test. ^ using Mann–Whitney U test. NA: Not applicable.

## Data Availability

The data that support the findings of this study are available from [Rana Abu-Farha] upon reasonable request due to privacy/ethical restrictions.
